# Development and Implementation of Short Courses to Support the Establishment of a Prehospital System in Sub-Saharan Africa: Lessons Learned from Tanzania

**DOI:** 10.1155/2019/3160562

**Published:** 2019-12-01

**Authors:** Hendry R. Sawe, Juma A. Mfinanga, Samwel Kisakeni, Patrick Shao, Paulina Nkondora, Libby White, Christina Bollinger, Irene B. Kulola, Upendo N. George, Michael S. Runyon, Erin Noste

**Affiliations:** ^1^Emergency Medicine Department, Muhimbili University of Health and Allied Sciences, Dar es Salaam, Tanzania; ^2^Emergency Medicine Department, Muhimbili National Hospital, Dar es Salaam, Tanzania; ^3^Emergency Medicine Association of Tanzania, Dar es salaam, Tanzania; ^4^Alfred Health, Melbourne, Australia; ^5^Department of Emergency Medicine, Atrium Health, Charlotte, NC, USA; ^6^Department of Emergency Medicine, University of California, San Diego, CA, USA

## Abstract

**Background:**

Tanzania has no formal prehospital system. The Tanzania Ministry of Health launched a formal prehospital system to address this gap. The Muhimbili University of Health and Allied Sciences (MUHAS) was tasked by the Ministry of Health to develop and implement a multicadre/provider prehospital curriculum so as to produce necessary healthcare providers to support the prehospital system. We aim to describe the process of designing and implementing the multicadre/provider prehospital short courses. The lessons learned can help inform similar initiatives in low- and middle-income countries.

**Methods:**

MUHAS collaborated with local and international Emergency Medicine and Emergency Medical Services (EMS) specialists to form the Emergency Medical Systems Team (EMST) that developed and implemented four short courses on prehospital care. The EMST used a six-step approach to develop and implement the curriculum: problem identification, general needs assessment, targeted needs assessment, goals and objectives, educational strategies, and implementation. The EMST modified current best EMS practices, protocols, and curricula to be context and resource appropriate in Tanzania.

**Results:**

We developed four prehospital short courses: Basic Ambulance Provider (BAP), Basic Ambulance Attendant (BAAT), Community First Aid (CFA), and EMS Dispatcher courses. The curriculum was vetted and approved by MUHAS, and courses were launched in November 2018. By the end of July 2019, a total of 63 BAPs, 104 BAATs, 25 EMS Dispatchers, and 287 CFAs had graduated from the programs. The main lessons learned are the importance of a practical approach to EMS development and working with the existing government cadre/provider scheme to ensure sustainability of the project; clearly defining scope of practice of EMS providers before curriculum development; and concurrent development of a multicadre/provider curriculum to better address the logistical barriers of implementation.

**Conclusion:**

We have provided an overview of the process of designing and implementing four short courses to train multiple cadres/providers of prehospital system providers in Tanzania. We believe this model of curricula development and implementation can be replicated in other countries across Africa.

## 1. Background

Low-income countries suffer the highest rate of injuries, infections, acute complications of pregnancy, and acute complications of communicable diseases including tuberculosis, malaria, and HIV [1]. Injury remains the single leading cause of morbidity and mortality in low- and middle-income countries (LMICs). The disproportionate burden of injury is evident in World Health Organization (WHO) data, demonstrating that about ninety percent of trauma-related deaths occur in LMICs. Furthermore, the majority of trauma deaths and maternal mortality in LMICs occur in the prehospital setting, before patients are able to reach stabilization or definitive treatment [2]. Prehospital systems in high-income countries reduce morbidity and mortality of emergent medical and trauma cases [3, 4]. However, most LMICs lack formal prehospital care systems and most of the population have no access to the most basic form of prehospital emergency care services [5, 6].

Tanzania, like most of LMICs, has no formal prehospital system. Patients access medical facilities through different mechanisms, including aid of a good Samaritan or bystander, police vehicles, private cars, or public transport [7]. In 2010, the first public emergency department (ED) opened at the Muhimbili National Hospital (MNH) in Dar es Salaam, providing an opportunity for acute resuscitation and stabilization of acutely ill and injured patients from across Tanzania. This was followed by the launch of specialist training in emergency medicine at the Muhimbili University of Health and Allied Sciences [8]. Within 2 years of starting emergency medicine training, hospital-wide mortality at Muhimbili National Hospital decreased by 40% [9]. While significant improvements in patient care and outcomes have been seen, the lack of a prehospital care system likely inhibits the potential magnitude of this success by delaying access to definitive care.

Developing and sustaining an effective prehospital system is complex and can be a resource intensive undertaking that requires input, support, and capital from multiple stakeholders. To address this need, the MoHCDGEC of Tanzania is piloting a prehospital system along the southern road corridor of Tanzania with the ultimate goal of replicating and expanding this model throughout the country [10]. This article describes the design and implementation of prehospital training courses in Tanzania.

## 2. Methods

We followed the six-step approach to curriculum development described by Kern [11]. Our manuscript provides an overview of the six steps of curriculum development and implementation for prehospital training courses in Tanzania.

### 2.1. Step 1: Problem Identification

Prior to our curriculum and training development, Tanzania had no formal prehospital training or formal EMS cadre/providers. Because of the government mandate and their commitment to supplying prehospital provider trainees, the first class of prehospital providers needed to be selected from the current government service scheme. Since prehospital clinicians do not currently exist within that scheme, there was a deliberate decision to train nurses, physicians, and drivers to provide prehospital care. This eliminated the need to identify and recruit trainees from outside of the current national healthcare system and allowed the trainees to continue to work for, and be paid by, the Ministry of Health.

### 2.2. Step 2: Targeted Needs Assessment and Stakeholder Consultative Meetings

Under the direction of the EMST, the EMAT and MUHAS conducted a needs assessment survey of a randomly selected group of general members, potential trainees, policymakers at the MoHCDGEC, and members of private ambulance services. The survey assessed the respondents' opinions on necessity of EMS training, appropriate timelines, the best modality of training, and whether an EMS-training course could be sustainable. A total of 100 randomly selected participants were interviewed: of these, 25 were government ambulance workers, 10 private ambulance workers, 30 students, 30 community members, and 5 members of MoHCDGEC.

The EMST met with relevant government agencies to discuss prehospital staffing since there is no official prehospital provider cadre in Tanzania. Given the different backgrounds and roles current government service workers would be expected to play, the EMST recommended the development of multicadre/provider curricula to include community first aid responders, fire rescue personnel, ambulance drivers, police officers, and ambulance providers. Additionally, the stakeholders recommended that the initial ambulance providers to be drawn from the ranks of existing government staff to allow for providers with a medical training background and official rank within the government to work in the EMS system while the position of paramedic is created in the government.

### 2.3. Step 3: Goals and Objectives

EMD-MUHAS and MNH signed a memorandum of understanding with the Emergency Medicine Association of Tanzania, and the two organizations collaborated to form the EMS Task Force (EMST) of expert physicians, nurses, and allied health providers to develop scope of practice, curriculum, and training for Tanzanian prehospital personnel. Among the members of the EMST were international collaborators from the African Federation for Emergency Medicine [12], the Alfred Hospital in Australia, and Carolinas Medical Center in the United States of America who had prior education, training, and experience in emergency medical systems (EMS).

Before specific learning objects and course development took place, the EMST met to determine the scope of practice and the prehospital protocols. The scope of practice and protocols drove curriculum development.

We defined scope of practice based on previous knowledge, skills, and training of the participants, the length of time allotted for the short-course curriculum, and the scope of practice design and modification based on available resources (e.g., medications, IV fluids, and oxygen). The protocols were developed with consideration of projected common illnesses and injuries as well as concern for possibility of MCI and highly infectious diseases, projected transport time and receiving facility resources and capabilities, and the prioritization of EMS provider safety, modifying protocols for specific scene safety concerns in Tanzania (e.g., protocols for responding to a call on a game reserve in the presence of wild animals).

The EMST and international collaborators developed a plan for two different types of curricula: short courses and a 3-year paramedic diploma. Four short courses were created: community first aider (CFA) for lay first responders; basic ambulance attendant (BAAT) for drivers, police officers, military, health attendants, and fire brigade personnel; basic ambulance provider (BAP) for clinicians and nurses; and the EMS dispatcher for telephone operators.

The EMST proposed a 3-year diploma course to train a more advanced prehospital provider and to create an official cadre/provider level of paramedics in Tanzania. The impact of paramedics or advanced life support (ALS) prehospital providers in low-income countries is not known. The OPALS study did demonstrate an improvement in mortality for respiratory distress patients but did not show an improvement of trauma patients [13]. More research is needed in the utility of ALS prehospital providers in the low-resource setting.

### 2.4. Step 4: Educational Strategies (Curriculum Development)

The development of short course curricula was a systematic process that started from the most advanced scope of practice (BAP) and then tailored the curriculum content to the needs of BAAT and CFA. This allowed for BAPs to serve as trainers for future short courses in BAAT and CFA. The international EMS specialist collaborators developed a training of the trainer curricula, to provide EMS training, skills, and knowledge to course instructors.

The CFA course goals are to create a cohort of lay providers who will be the first line of care, activate the prehospital system by calling the dispatch center, and serve as community advocates for the newly established prehospital system. The EMST engaged with the Tanzanian Red Cross Society (TRCS) [14], whose first aid course content is based on the African First Aid materials [15]. This partnership enabled MUHAS and TRCS to harmonize their curriculum and training content. The content level of CFA is comparable to that of the basic ambulance assistant level and first aid course in South Africa and the United States, respectively [16, 17].

BAAT training advances from the CFA and is tailored towards designated first responders (including police, fire rescue, ambulance drivers, and military personnel). In Tanzania, first responders are public safety agency personnel and are expected to respond to a scene of injury or illness [7, 9]. They do not have any formal medical training. Training of first responders in other LMIC countries has to be feasible and effective in a setting with a limited or absent prehospital systems [18, 19]. The BAAT training is similar to the Emergency Medical Responder training in the USA [17].

The dispatch unit will be centralized at the Muhimbili National Hospital [20] for the EMS pilot phase. For the initial phase, eligible candidates are telephone operators and information technology specialists working at MNH. The EMS dispatcher training was developed last, taking into consideration the communication, clinical, and facility logistics.

The paramedic diploma course is still in the curriculum development phase with planned first enrollment for October 2020.

### 2.5. Step 5: Implementation

MUHAS approved the four short courses' curricula, and training started in October 2018 and finished in July 2019. The training of the BAPs and BAATs started first while the training of the trainers for Community First Aid took place. Finally, the EMS Dispatcher training was held in May 2019–June 2019.

### 2.6. Step 6: Evaluation and Feedback

Evaluation, feedback, and continuous improvement of the curriculum occurred throughout all stages of curriculum design, piloting of course material, administration of the courses, and postcourse review process. The curriculum went for formal review to the MUHAS review committee for curriculum and provided feedback to the EMST.

During piloting and administration of the courses, we used participant feedback for improvement. This was achieved by precourse and postcourse surveys, prewritten and postwritten course exams, skill station exam results, and an after-action review process of the cohort with the EMST and course instructors.

The evaluations, feedback, and results of the four short courses will be used as the EMST develops the curriculum for the paramedic diploma. A refresher course will occur for the BAPs, BAATs, and EMS Dispatcher within 1 month of the EMS pilot starting. The refresher course will have precourse and postcourse examinations and surveys for further evaluation, feedback, and assessment of knowledge retention.

## 3. Results

### 3.1. Steps 1 and 2: General and Targeted Needs Assessment

The response rate for the survey was 100%, and we found an overwhelming support (95.7%) towards establishing formalized EMS provider training in Tanzania. All of the policymakers at the MoHCDGEC felt that prehospital training is necessary and fully supported the training. Forty four percent of survey participants thought prehospital training should be 3 or less months, while 16% thought it should be more than 1 year in length (Table 1).

### 3.2. Steps 3 and 4: Goals, Objectives, and Educational Strategies (Curriculum Development)

The team developed the core content for the four short courses after consultation from stakeholders and experts and development of EMS scope of practice (Table 2). The team created training manuals, presentation slide decks, skill stations, case scenarios, and examinations for each of the courses.

The courses were divided into three main learning methodologies: lecture, hands-on skill stations, and field experience in the EMD and maternity ward. Prehospital services do not exist currently in Tanzania; therefore, field experience was limited to the EMD and maternity ward as there were no available ambulance services for the students to participate in “ride time.”

### 3.3. Step 5: Implementation

The official training launched in the November of 2018 and finished in July 2019. The team trained 63 BAPs, 104 BAATs, 25 EMS Dispatchers, and 287 CFAs. The overall short course pass rate was 90.6% (Table 3). Course pass rate was based on course attendance, hands-on skills exams, and written exam.

### 3.4. Step 6: Evaluation and Feedback

Evaluation and feedback occurred throughout the development and delivery of the short courses. The MUHAS review committee gave specific feedback on course eligibility, content, and duration, and the EMST adapted the curriculum before piloting the training. In June 2018, the EMST conducted the first pilot training to test the course content and certify the trainers through this process. The results of the pilot training allowed for course content modification. The pilot class had a strong grasp on theoretical knowledge but required increased time to successful demonstrate skill mastery. Therefore, a critical modification was to increase the time dedicated to hands-on skill stations. Slides were also edited to directly reference the ambulance provider protocols. The modification changed the time spent for hands-on skills and field experience to 60% for the BAP (Table 4).

Modifications to the curriculum were made during the course in order to achieve the set objectives for the courses. An example of course modification was adding more time to c-spine immobilization and patient movement after receiving feedback during the weekly meeting that course participants needed more practice to master these skills.

Overall, participants improved on their written tests. Both the BAPs and BAATs improved on their written tests and averaged a passing score on their skills exam (Table 5). Written feedback was obtained and details of individual course evaluation, content comfort, and feedback. Overall, participants rated their comfort level with prehospital skills significantly improved in comparison to their precourse survey (Table 6).

## 4. Discussion

Emergency care in Tanzania has improved significantly in the last decade [21]. Despite these advancements, patients often present to hospitals at late stages of illness and injury. We believe that a trained, equipped, and organized prehospital system will improve access to emergency services and ultimately save lives in Tanzania. The EMS development project focused on multicadre prehospital care providers, defining the scope of practice of the prehospital providers, curricula development, and training.

In Tanzania, there is no formal prehospital system, no cadre/provider level designated to serve prehospital care in the government, and there is no formal training towards prehospital level providers [7]. During the process of designing and implementing this training, several items were considered: the working environment; appropriate entry level and skills to be attained; the post training cadre/provider level recognition; and, advancement options.

Successful development of four short courses for prehospital care was achieved by a practical approach to the problems and restrictions of the pilot project. Our manuscript provides an outline of the design and implementation of several curricula for prehospital care providers in Tanzania. Prehospital care in low-resource settings is limited [5, 6]. Previous studies describe the training of lay first responders and do not include the concurrent development of curriculum training of multiple different levels of prehospital responders [17, 18, 20, 22]. The diversity and magnitude of the curricula package designed is unique, and the first to be described in Sub-Saharan Africa (SSA), to the best of our knowledge.

We conducted curricula needs assessment with different stakeholders, the results of which revealed an overwhelming support towards the development and establishment of prehospital training. This multistakeholder needs assessment resulted in buy-in for the prehospital program. The MoHCDGEC stakeholders supported, and emphasized, the need to initiate the process of concurrent development of a prehospital cadre/provider level in the government service scheme in parallel with the trainings, to ensure availability of positions for employment upon graduation. This is an important consideration of any LMIC prehospital curricula development, as it has been shown in other SSA settings that integration of prehospital personnel into existing public or private schemes of service is a critical factor in ensuring sustainability [22, 23].

The uniqueness of our prehospital provider trainee cohort (with the exception of the CFA cohort) is that all are currently employed in the public sector, serving as clinicians, nurses, health attendants, ambulance drivers, or police/fire rescue personnel. This will ensure the sustainability of the knowledge received, as all of the providers will return to the same workstations that have been earmarked for the EMS pilot project. Provider task shifting after short course trainings has been shown to have impact in a wide range of in-hospital clinical practice [18, 19].

The scope of practice of the different levels of prehospital providers was defined prior to curriculum development and modified to fit the Tanzanian context. This was imperative to the process of creating curricula that matched the local context and resources.

Previous studies have described curriculum and program development in LMICs with a single prehospital provider cadre [24]. It was important for our system development and education to develop additional related courses to meet the practical needs of a prehospital pilot project that will rely on community first responders, police officers, fire personnel, drivers, and ambulance providers.

Our current success is due to several key factors: the multidisciplinary input and support from national leaders; a proper needs assessment; formation of the EMST to include Tanzanian emergency physicians and nurses who are experts on emergency care delivery in Tanzania; consultation by international collaborators with knowledge and education in EMS and experience working in Tanzania; modifying EMS best practices to the Tanzanian context of emergency care in a low-resource setting; and, piloting and modifying training to fit the skills and needs of the prehospital students.

Inevitably this process encountered some challenges, notably the lack of in-country EMS experts, which necessitated significant time and investment of international collaborators to support the design and testing of the curricula as well as mentor local experts to ensure sustainability.

## 5. Conclusion

This article has provided an overview of the process of designing and implementing four short courses to train multiple cadres of prehospital system providers in Tanzania. We believe this model of curriculum development and implementation can be replicated in other countries across Africa and in other LMICs.

## Figures and Tables

**Table 1:** Summary of the findings from targeted survey.

Participants (*N* = 100)	*n*	%
Government ambulance staff	25	25
Private ambulance staff	10	10
Secondary school students	30	30
Community members	30	30
MoHCDGEC policymakers	5	5

Working or worked on ambulance (*N* = 100)	35	35

EMS training course necessity (*n* = 100)	67	95.7

Proposed duration of the course	*N* = 57	%
6 weeks	14	24.6
3 months	11	19.2
6 months	2	3.5
1 year	15	26.3
3 years	9	15.8
Unknown	6	10.5

Potential course participants	*n*/*N*	%
Willingness to participate in the course	54/65	83.1
Proposed course model: full time	21/55	38.2
Proposed course model: part time	34/55	61.8
Modality of course payment: self	18/54	33.3
Modality of course payment: government	11/54	20.4
Modality of course payment: others	25/54	46.3

Policymakers		
Support formalized training programme	5/5	100

**Table 2:** Course length and core curriculum content for Tanzania EMS short courses.

	Basic ambulance provider	Basic ambulance attendant	Community first aid	Emergency medical services dispatcher
Length of course	6 weeks	4 weeks	5 days	2 weeks

Core content	Introduction to the EMS profession	Introduction to prehospital care	First aid basics	Community first aid
	Overview of basic anatomy, physiology, and pharmacology	Driving safety and emergency traffic rules	Cardiac and choking emergencies	Introduction to EMS dispatch
	Basic life support and the “ABC's”	Scene safety, lifting, and patient movement	Common illnesses and injuries	Telecommunication and communication essentials
	Patient assessment	Daily ambulance checklist	Patient movement, transport, and transfer	EMS dispatch stress
	Prehospital trauma care	Infection prevention control		Basic medical dispatch
	Medical emergencies	Introduction to EMS dispatch		EMS dispatch protocols
	Transport operations, vehicle extrication, special rescue	Basic life support and the “ABCs”		Dispatch life support
	Psychiatric emergencies	Haemorrhage control		
	Environmental emergencies and toxicology	Incident management, Mass causality incidents, and triage concepts		
	Paediatrics	Environmental emergencies and toxicology		
	Obstetrics and neonatal care	Psychiatric emergencies		
	Incident management, Mass causality incidents, and triage concepts			

**Table 3:** Number trained and pass rate for Tanzania EMS short courses.

Short course	Number trained	Number passed (%)
Basic ambulance provider	62	62 (100)
Basic ambulance attendant	104	98 (94.2)
Community first aid	287	251 (87.5)
EMS dispatcher	25	22 (88)
Total	478	22 (90.6)

**Table 4:** The distribution of time in the classroom and skills stations/field experience.

Course name	Lecture (%)	Practical^*∗*^ (%)
Basic ambulance provider (BAP)	40	60
Basic ambulance attendant (BAAT)	45	55
Community first aid (CFA)	30	70
Emergency medical services dispatch	50	50

^*∗*^Skills stations or field experience.

**Table 5:** BAP and BAAT pre/postcourse results.

	Precourse written mean score	Postcourse written mean score	*P* value
Basic ambulance provider	57% (SD 9.2)	74% (SD 9.8)	<0.0001
Basic ambulance attendant	56% (SD 14)	79% (SD14)	<0.0001

**Table 6:** BAP and BAAT participant-rated prehospital skills comfort level, before and after course.

Short course	Skills	Precourse mean	Postcourse mean	*P* value
Basic ambulance provider	Scene safety	2.8 (SD 0.6)	4.9 (SD 0.05)	<0.0001
	Patient movement	3.0 (SD 0.3)	4.9 (SD 0.1)	<0.0001
	Communication	3.2 (SD 0.05)	4.8 (SD 0.2)	<0.0001
	Primary survey	2.7 (SD 0.6)	5.0 (SD 0)	<0.0001
	Airway adjuncts	2.7 (SD 0.4)	4.9 (SD 0)	<0.0001
	Bag-valve mask	2.4 (SD 0.4)	4.9 (SD 0)	<0.0001
	AED	2.5 (SD 0.98)	4.7 (SD 0.1)	<0.0001
	Patient restraints	2.4 (SD 0.7)	4.8 (SD 0.1)	<0.0001
	C-spine immobilization	2.6 (SD 0.5)	4.8 (SD 0.05)	<0.0001
	Obstetric delivery	3.3 (SD 0.2)	4.8 (SD 0.05)	<0.0001

Basic ambulance attendant	Scene safety	3.2 (SD 0.4)	4.9 (SD 0.1)	<0.0001
	Patient movement	3.2 (SD 0.2)	4.9 (SD 0.1)	<0.0001
	Head tilt, chin lift	1.9 (SD 0.6)	4.6 (SD 0.2)	<0.0001
	Bag-valve mask	1.9 (SD 0.3)	4.6 (SD 0.3)	<0.0001
	CPR adult	2.1 (SD 0.3)	4.8 (SD 0.2)	<0.0001
	AED	1.5 (SD 0.3)	4.5 (SD 0.3)	<0.0001
	Patient restraints	2.4 (SD 0.4)	4.5 (SD 0.3)	<0.0001
	Bleeding control	2.7 (SD 0.2)	4.9 (SD 0.1)	<0.0001
	C-spine immobilization	1.8 (SD 0.4)	4.5 (SD 0.5)	<0.0001
	Driving emergency traffic	3.1 (SD 0.3)	4.7 (SD 0.1)	<0.0001

^*∗*^Scale: 1 = 5 = strong comfort or knowledge; 4 = comfort or knowledge; 3 = some comfort or knowledge; 2 = minimal comfort or knowledge; 1 = no comfort or knowledge.

## Data Availability

The dataset supporting the conclusions of this article is available from the authors on request.

## Abbreviations

BAAT: Basic ambulance attendant
BAP: Basic ambulance provider
CFA: Community first aid
DCEPD: Directorate of continuing professional development
EMAT: Emergency medicine association of Tanzania
EMS: Emergency medical services
MNH: Muhimbili National Hospital
MUHAS: Muhimbili University of Health and Allied Sciences.
